# A novel RNA binding surface of the TAM domain of TIP5/BAZ2A mediates epigenetic regulation of rRNA genes

**DOI:** 10.1093/nar/gkv365

**Published:** 2015-04-27

**Authors:** Irina Anosova, Svitlana Melnik, Konstantinos Tripsianes, Fatiha Kateb, Ingrid Grummt, Michael Sattler

**Affiliations:** 1 Institute of Structural Biology, Helmholtz Zentrum München, Neuherberg D-85764, Germany; 2 Biomolecular NMR and Center for Integrated Protein Science Munich (CIPSM), Department Chemie, Technische Universität München, Garching D-85747, Germany; 3 Division of Molecular Biology of the Cell II, DKFZ-ZMBH Alliance, German Cancer Research Center, Heidelberg D-69120, Germany

## Abstract

The chromatin remodeling complex NoRC, comprising the subunits SNF2h and TIP5/BAZ2A, mediates heterochromatin formation at major clusters of repetitive elements, including rRNA genes, centromeres and telomeres. Association with chromatin requires the interaction of the TAM (TIP5/ARBP/MBD) domain of TIP5 with noncoding RNA, which targets NoRC to specific genomic loci. Here, we show that the NMR structure of the TAM domain of TIP5 resembles the fold of the MBD domain, found in methyl-CpG binding proteins. However, the TAM domain exhibits an extended MBD fold with unique C-terminal extensions that constitute a novel surface for RNA binding. Mutation of critical amino acids within this surface abolishes RNA binding *in vitro* and *in vivo*. Our results explain the distinct binding specificities of TAM and MBD domains to RNA and methylated DNA, respectively, and reveal structural features for the interaction of NoRC with non-coding RNA.

## INTRODUCTION

**Figure 1.**
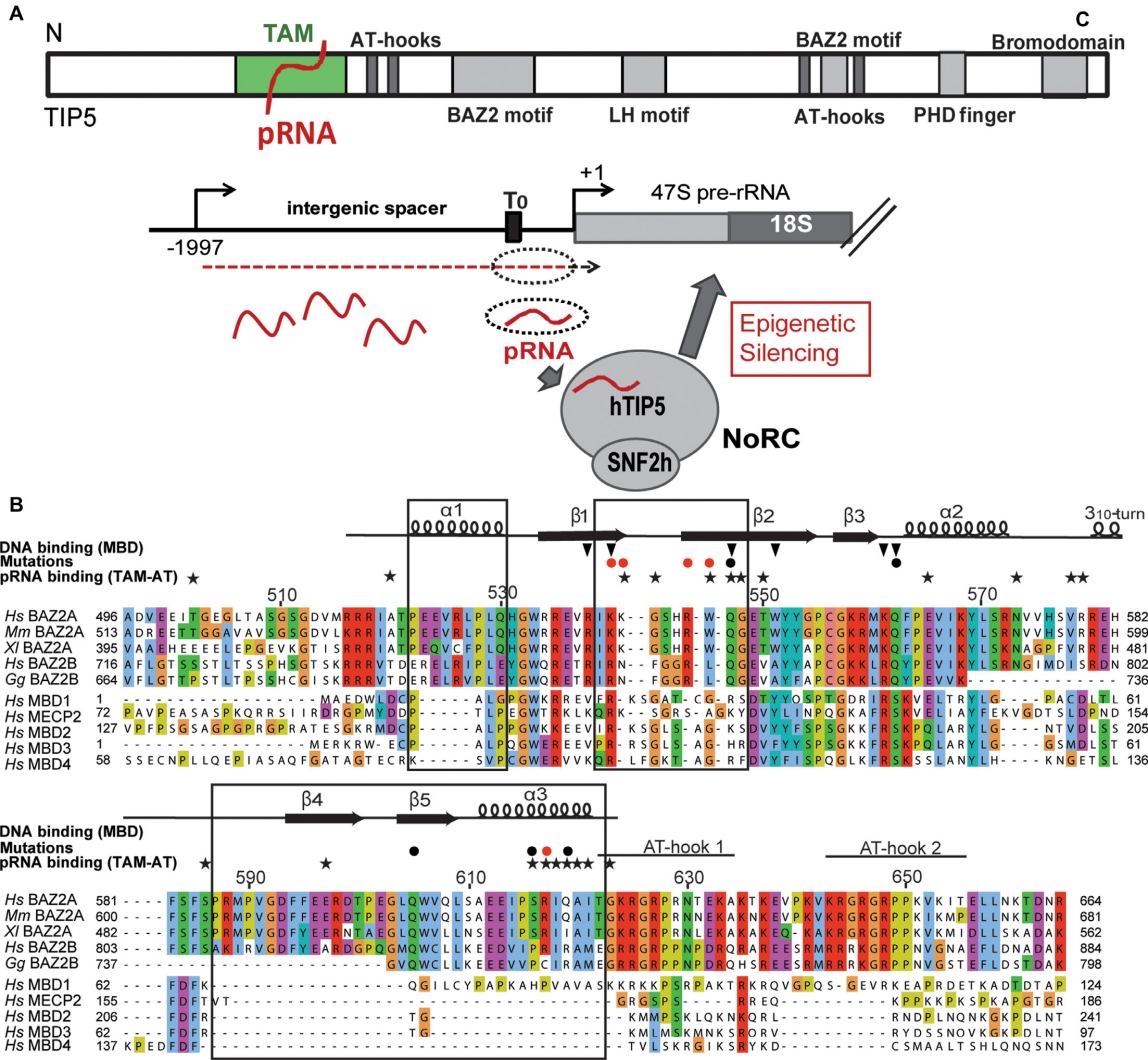
Domain organization of TIP5 and its TAM domain. (**A**) Top: domain organization of human TIP5. Bottom: the role of pRNA in NoRC-mediated silencing of rRNA genes (1). Intergenic transcripts (red dashed line) originating from the rDNA intergenic spacer are processed into 150–250 nt transcripts, termed pRNA (red waved lines). The interaction of pRNA with TIP5 guides NoRC to the rDNA promoter, an essential step in NoRC-dependent silencing of rRNA genes. (**B**) Multiple sequence alignment of the TAM domain of hTIP5 (BAZ2A) with phylogenetically related BAZ2A homologs (14) and canonical human MBDs. Sequences were aligned using MUSCLE algorithm (47), color coding according to CLUSTAL-X (48). Secondary structure observed in human TAM (Figure 3) is indicated above the sequence. Structural features of the TAM domain that are not present in MBD folds are indicated by black boxes. Black stars mark residues that are strongly affected in NMR titrations of TIP5/TAM-AT with pRNA^mini^. Red and black filled circles indicate residues that were mutated to probe the TAM/pRNA interaction interface showing strong or no effect on RNA binding, respectively. Black triangles indicate key residues of the MBD1 that are crucial for its interaction with methylated CpG (52). Many of these residues are not conserved in TAM domains, i.e. TIP5 TAM R538, Q547, Q562. Residue numbering refers to human TIP5.

Despite the fact that mammalian cells contain several hundreds of rRNA genes (rDNA) and rRNA genes are the most actively transcribed genes in eukaryotes, a large fraction of rDNA is silenced by heterochromatin formation to prevent aberrant homologous recombination, thus safeguarding rDNA stability and nucleolar integrity (1). The transcriptionally silent state of rDNA is established by the chromatin remodeling complex NoRC, which comprises the DNA-dependent adenosine triphosphatase SNF2h and a large subunit, termed TIP5 (also known as BAZ2A) (2) (Figure 1a). The C-terminal part of TIP5 harbors a tandem PHD finger/bromodomain, a cooperative unit that interacts with chromatin modifying enzymes, which set specific heterochromatic histone marks and trigger *de novo* DNA methylation (3,4). Targeting NoRC to rDNA leads to repositioning of the promoter-bound nucleosome, changes in histone modifications, increased DNA methylation and silencing of rRNA genes (5–8). Consistent with epigenetic regulation representing an intimate and balanced interplay of both RNA and chromatin fields, NoRC function requires the association of TIP5 with specific 150–250 nt RNA, named pRNA (‘promoter-associated RNA’) as its sequence overlaps the rDNA promoter (6,9). pRNA folds into a phylogenetically conserved hairpin structure that is recognized by TIP5 and this specific secondary structure of the pRNA is required for guiding NoRC to nucleoli (10). Mutations that disrupt this specific stem-loop structure impair binding of TIP5 to pRNA and abolish the nucleolar localization of NoRC,whereas compensatory mutations that restore the hairpin structure re-establish binding and targeting NoRC to nucleoli. The interaction with pRNA is mediated by the TAM (TIP5/ARBP/MBD) domain in the N-terminal region of TIP5, and is indispensable for nucleolar localization of NoRC and heterochromatin formation at rDNA. RNase footprinting and protease sensitivity experiments suggest that TIP5 associates with pRNA in an induced fit mechanism, resulting in structural changes that may facilitate the interaction with co-repressors to promote heterochromatin formation and rDNA silencing (6). A recent report demonstrated that the poly (ADP-ribose)-polymerase-1 (PARP1) associates with TIP5, thus acting as a co-repressor that mediates the inheritance of silent histone marks through cell cycle progression (11). Significantly, NoRC function is not restricted to rDNA silencing, but it has been shown to also establish a repressive heterochromatic structure at centromeres and telomeres, thus preserving the structural integrity of these repetitive genomic loci (12). Paradoxically, TIP5 may serve as a useful marker for the metastatic potential in prostate cancer. By interacting with EZH2, TIP5 hypermethylates and silences genes that are repressed in metastasis (13).

The TAM domain in the N-terminal region of TIP5 exerts sequence homology to methyl-CpG-binding domains (MBDs), which are found in proteins that bind to methylated DNA and mediate methylation-dependent gene silencing (14). TIP5 also harbors two AT-hooks C-terminally of the TAM domain, which are known to bind DNA and regulate transcription by modifying the architecture of DNA (15). Canonical MBDs bind to methylated cytosine in the context of CpG motifs in DNA but do not directly bind RNA (16). The closely related TAM domain of TIP5, on the other hand, directly contacts pRNA and this interaction is essential for targeting NoRC to rDNA and NoRC-dependent silencing of rRNA genes (10).

Here, we report the three-dimensional (3D) solution structure of the TAM domain of human TIP5 (hTIP5). We show that the TAM domain represents a variant of the MBD fold with unique C-terminal extensions that are required for the interaction of TIP5 with pRNA. Using biochemical studies and NMR-spectroscopy we show that point mutations in this surface impair RNA binding *in vitro* and *in vivo*. Our results provide structural details for interactions between TIP5 and RNA that are essential for recruitment of the chromatin remodeling complex NoRC to specific genomic loci.

## MATERIALS AND METHODS

### Plasmid constructs, cells, general procedures

HEK293T cells were grown in DMEM (Invitrogen) supplied with 10% fetal calf serum (FCS; Biosera), 11 mg/ml sodium pyruvate and 11 mg/ml penicillin and streptomycin (PAA Laboratories GmbH). DNA encoding human TIP5/TAM-AT (residues 496–664) and human TIP5/TAM (residues 516–623) were subcloned from pJC40-hTIP5(N332–723) using standard cloning protocols into modified pETM-11 vector (EMBL) containing a His_6_-tag for purification, a GB1-tag at the N-terminus for protein solubility and a tobacco etch virus cleavage site for removal of the tags. TIP5 variants were obtained by QuickChange site-directed mutagenesis (Agilent Technologies). pJC40-hTIP5(N332–723) and pJC40-mTIP5(N1–598) used for purification of human or murine TIP5 for *in vitro* studies were described before (6). HA-FLAG-hTIP5 containing full-length human TIP5 or TIP5 mutants used for *in vivo* experiments was described before (2). pTOPO2.1-mrDNA (-204/-1 sense) used for the synthesis of mouse pRNA was described before (6).

### Protein expression and purification

Isotopically labeled proteins for NMR studies were produced by overexpression in *Escherichia coli* BL21 (DE3) cells in minimal medium (M9) enriched with ^15^N, ^13^C and/or ^2^H, supplemented with 50μg/ml kanamycin. ^15^N/^13^C- and ^15^N-labeled proteins were prepared using M9 medium with [U-^13^C]-D-glucose (2g/l) and/or ^15^NH_4_Cl (1g/l) as sole carbon and nitrogen sources. For 50% random fractional deuteration the M9 medium was based on 90% ^2^H_2_O. Media were inoculated overnight and grown to OD_600_ = 0.8 at 37°C. Expression was induced with 0.2 mM IPTG and performed at 18°C for at least 16 h.

*Escherichia coli* transformed with cDNA encoding wild-type or mutant TIP5 were resuspended in lysis buffer containing 50 mM HEPES [pH 8.0], 1 M NaCl, 10 mM imidazole, 2 mM β-mercaptoethanol, 1 mM Pefabloc SC (Serva), 1 μg/ml lysozyme and 7μg/ml DNase I, 0.2% w/v IgepalCA-630. After sonication and high-speed centrifugation, His-tagged proteins were bound to Ni-NTA Agarose (Quiagen). Insoluble TIP5/TAM was purified from the pellet by resuspending in low salt lysis buffer with 6 M guanidinium hydrochloride and refolding on a Ni^2+^ column by washing with decreasing concentrations of guanidinium hydrochloride (2–0 M). After washing with low salt and high salt buffer containing 1.8 M NaCl, bound proteins were eluted with 300 mM imidazole, digested with tobacco etch virus protease (1 μg/mg protein at 4°C overnight). The tags were removed with a second Ni^2+^column and recombinant proteins were further purified by size-exclusion chromatography (HiLoad 26/60 Superdex 75, GE Healthcare) in low salt lysis buffer. For NMR measurements proteins were kept in 20 mM sodium phosphate [pH 7.0], 50 mM NaCl, 1 mM Dithiothreitol (DTT), 10% v/v ^2^H_2_O.

### RNA transcription and complex formation

pRNA^mini^ was transcribed *in vitro* by T7 RNA polymerase from a double stranded synthetic DNA template (17,18) using 12 mM MgCl_2_, 4 mM adenosine triphosphate (ATP) and cytidine triphosphate (CTP), 4.8 mM guanosin-5'-triphosphate (GTP) and uridine-5'-triphosphate (UTP) and 0.08mg/ml PEG8000 (Promega). For reduction of *n* + 1 products the bottom strand was methoxylated at two 5′-terminal nucleotides (19). Transcripts were precipitated with ethanol, purified by preparative 20% (19:1) PAGE and electroeluted at 4°C. Residual acrylamide was removed by dialysis in 10 mM sodium phosphate [pH 6.0], 0.02 mM ethylenediaminetetraacetic acid (EDTA), 0.02 mM NaN_3_ at 1-, 0.5- and 0 M NaCl. Formation of the stem-loop in solution was induced by denaturing pRNA^mini^ in NMR buffer for 4 min at 95°C and snap-cooling on ice for 15 min and controlled by NMR.

Complex formation was performed at low concentrations on ice. The required amount of pRNA^mini^ in 1.5 ml of cold NMR buffer was slowly added to 33 nmol protein in 2.5 ml of cold NMR buffer. The solution was gently mixed, left for 20 min on ice to allow complex formation and concentrated in an Amicon^®^ Ultra centrifugal filter unit (Merck Millipore) to yield a complex of 0.15 mM TIP5/TAM-AT with molar equivalents of pRNA^mini^. NMR data were recorded on the complex in a Shigemi tube directly thereafter.

### NMR spectroscopy

NMR measurements were carried out at 298K (TIP5/TAM-AT) and 293K (TIP5/TAM) on Bruker 900-, 750- or 600 MHz spectrometers equipped with room temperature (750 MHz) or cryoprobes (900, 600 MHz) and pulsed field gradients. Spectra were processed with NMRPipe (20) and analyzed using Sparky 3.110 software (T. D. Goddard, D. G. Kneller, SPARKY 3, University of California, San Francisco). Backbone resonances were derived using a standard set of 3D NMR experiments for backbone assignment (21) (HNCA, HNCACB, HN(CO)CACB and CBCA(CO)NH) on ^15^N/^13^C uniformly labeled and in case of TIP5/TAM-AT 50% randomly deuterated protein samples. Secondary chemical shifts were calculated and used for secondary structure prediction as described previously (22,23). ^1^H and ^13^C side chain resonances were assigned based on 3D HCCH-TOCSY experiments with ^13^C and ^1^H evolution and correlated to amide group by CC(CO)NH-TOCSY. ^13^C*β* and ^1^H*δ/ϵ* of aromatic residues were correlated via scalar couplings (24). The tautomeric state of histidines was determined from long range ^1^H,^15^N-HMQC (25). Stereospecific assignments of pro-S and pro-R methyl ^1^H resonances of valine and leucine were based on a 10% ^13^C labeled NMR sample as described (26,27). Backbone- and side chain residues were assigned manually supported by the MARS software (28). NOE-assignments were performed using CYANA 3.0 (29) and checked manually. Torsion angle restraints were derived from TALOS+ (30).

^1^H^N^−^15^N and ^13^CO−^15^N RDC were recorded in a two-dimensional doublet-separated, sensitivity enhanced HSQC experiment (31). The samples were partially aligned using 7% PAA compressed gel (32) or 4% w/v 3:1 DMPC/DHPC bicelles (33) (Avanti Polar Lipids).

Relaxation measurements were performed at 900 MHz proton Larmor frequency on a 0.2 mM ^15^N-labeled sample of TIP5/TAM-AT at 298K and at 750 MHz proton Larmor frequency on a 0.6 mM ^15^N/^13^C labeled sample of TIP5/TAM at 293K. *R*_1_, *R*_1ρ_ and *R*_2_ were measured in an interleaved fashion as described (34). The *R*_2_ relaxation rate was derived from *R*_1ρ_ (35) for TIP5/TAM-AT and measured directly for the TIP5/TAM domain. NMR spectra were processed and analyzed with NMRPipe (20) using NMRDraw for visualization. Peak intensities were extracted at different delays from peak volume for non-overlapping signals. The relaxation rates were fitted to a 2-parameter exponential decay from the intensities, with errors from duplicate experiments, calculated from both the standard deviation and the fit itself. The {^1^H}-^15^N heteronuclear NOE was extracted as the ratio of peak intensities recorded with and without saturation. The error was estimated from intensity of experimental noise with error propagation. Analysis of the relaxation data was performed as described previously (36). The tumbling correlation time (*τ*_c_) was estimated from *R*_2_/*R*_1_ (37) and compared to the expected value, calculated from the number of residues in a monomer (38). Relaxation data were plotted using Xmgrace.

Proton/deuterium exchange data were measured at 600 MHz Larmor frequency on a 0.2 mM ^15^N-labeled sample at 293K. The protein sample was split in half, one being used for setting up experimental parameters on the spectrometer, the other being lyophilized and redissolved in 100% ^2^H_2_O immediately before measurement. The exchange was detected by the reduction/disappearance of the respective ^1^H^N^-^15^N signals in a series of SOFAST HMQCs (39) recorded at time intervals ranging from 8–120 min. The experiment was recorded with 8 scans, taking ca. 5–6 min per measurement.

NMR chemical shift perturbation experiments were measured at 600 MHz Larmor frequency on a complex of 0.15 mM ^15^N-labeled, 50% deuterated TIP5/TAM-AT with different molar ratios of unlabeled pRNA^mini^ at 298K. Titrations of TIP5/TAM with additional RNA and DNA constructs were measured on 0.1–0.15 mM ^15^N-labeled and ^15^N- and 10% ^13^C-labeled samples at 293 K and Larmor frequency of 600 MHz and 500 MHz, respectively. Nucleic acids were added at increasing molar ratios to at least equimolar amounts. Chemical shift changes of ^1^H^N^-^15^N signals of the protein were monitored in a series of ^1^H, ^15^N HSQC experiments. The peak positions were determined using the SPARKY 3.110 automatic picking algorithm. Chemical shift changes are calculated as:

\begin{equation*}
\Delta \delta _{ppm} = \sqrt {(\delta H_x - \delta H_0 )^2 + \left( {\frac{{\delta N_x - \delta N_0 }}{5}} \right)^2 } ,
\end{equation*}

where *δH*_x_ and *δN*_x_ are the respective chemical shift values of a resonance in the complex; *δH*_0_ and *δN*_0_ are the respective chemical shift values of the same resonance in the free protein. The factor 1/5 was used to account for the different ppm spectral range of proton and nitrogen spins. Resonances with chemical shift changes or intensity drops larger than one standard deviation above the average were considered significant for binding.

### Structure determination

The solution structure of the TIP5/TAM domain was defined based on 2732 distance restraints derived from CYANA 3.0 calculations, torsion angle restrains and two sets of RDCs measured in different alignment media. Two hundred structures were calculated and water-refined by ARIA1.2/CNS (40,41). The 20 lowest-energy structures were used for quality and structure validation by the iCING web interface (42), PROCHECK (43) and WHATCHECK (44). Backbone torsion angles for 92.1, 7.8 and 0.1% of all residues are found in the core, allowed and generally allowed regions of the Ramachandran plot, respectively, none in disallowed regions. The RDC R-factor was calculated as described previously (45). Electrostatic potential maps were calculated online using PyMOL (The PyMOL Molecular Graphics System, Version 1.7.4 Schrödinger, LLC). Structural representations were generated with PyMOL.

### Electrophoretic mobility shift assays (EMSA)

Electrophoretic mobility shift assays (EMSA) experiments were performed as described previously (6). Radiolabeled MCS-RNA was synthesized by T7 RNA polymerase using pBluescript-KS linearized with EcoRI and transcribed with T7 RNA polymerase in the presence of 50 μCi [^32^P]-UTP (3,000 Ci/mmol; Perkin Elmer). Radiolabeled RNA was incubated for 30 min on ice with purified recombinant protein (10–50 nM) in 12 mM Tris–HCl, pH 8.0, 100 mM KCl, 5mM MgCl_2_, 0.1 mM EDTA, 0.5 mM DTT, 3% glycerol. In competition experiments (Supplementary Figure S2), unlabeled competitor RNA was added after 5 min and reactions were incubated for additional 30 min. RNA–protein complexes were resolved in 6% native polyacrylamide gels in 0.5× Tris/Borate/EDTA buffer (TBE). Gels were vacuum-dried, exposed to a screen and visualized by PhosphorImaging. The amount of bound RNA in band shifts and unbound RNA probes was compared by quantification of images using Image Gauge 4.0 software.

### Filter binding assays (FBA)

Filter binding assays (FBA) were performed essentially as described (46).

**Figure 2.**
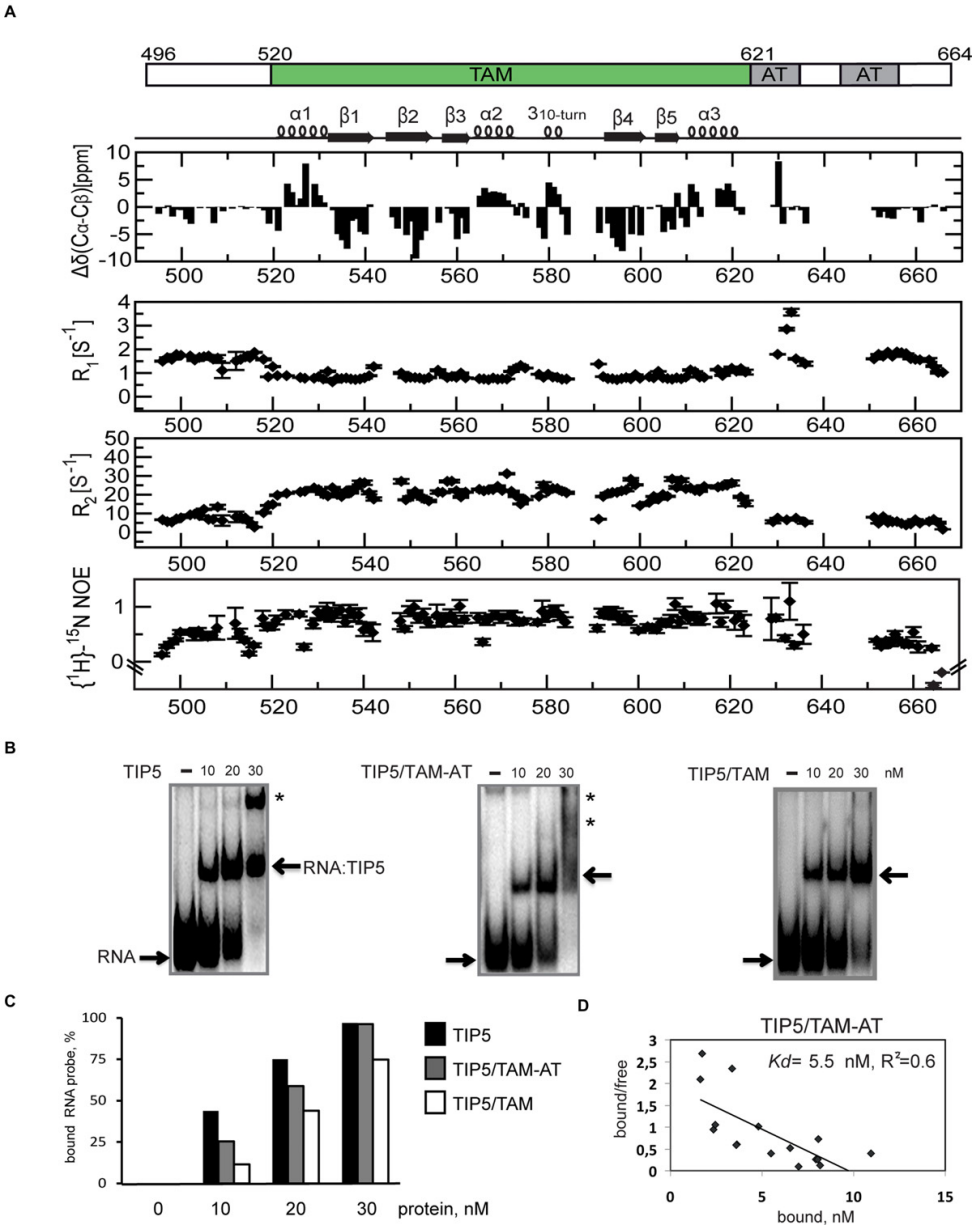
NMR and biochemical analysis of RNA binding by the TIP5/TAM domain. (**A**) NMR analysis of the TIP5/TAM-AT domain. ^13^C secondary chemical shifts Δδ(^13^C_α_) − Δδ(^13^C_β_), ^15^N *R*_1_, *R*_2_ relaxation rates and {^1^H}-^15^N heteronuclear NOE values are plotted versus TIP5 residue numbers. Secondary structure elements of the TAM domain are indicated on top. (**B**) The TAM-AT domain represents the minimal RNA binding region of TIP5. Electrophoretic mobility shift assays (EMSA) showing binding of TIP5, TIP5/TAM and TIP5/TAM-AT to RNA. 0, 10, 20 and 30 nM of the respective proteins were incubated with 1 nM of [^32^P]-MCS-RNA probe. Positions of free RNA probe and RNA:TIP5 complexes are marked with arrows. Super-shifted binding products are marked with asterisks. (**C**) TIP5/TAM-AT has almost the same RNA binding efficiency as TIP5. Amounts of bound RNA in the EMSAs shown in panel (B) were quantitated using the ImageGauge software and are presented as percentage of input probe. (**D**) Scatchard plot analysis of *in vitro* binding of TIP5/TAM-AT to pRNA. Binding kinetics to pRNA were monitored by filter binding assay. The dissociation constant (*K*_D_) was calculated using Scatchard plot analysis from the ratio of bound to bound/free probes. The resulting fit (*R*^2^ = 0.6) yields an RNA binding affinity constant for the TIP5/TAM-AT/pRNA interaction of *K*_D_ = 5.5 nM.

For synthesis of [^32^P]-labeled pRNA, pTOPO2.1-mrDNA(-204/-1) was linearized with EcoRI and transcribed with T7 RNA polymerase in the presence of 50 μCi [^32^P]UTP (3000 Ci/mmol; Perkin Elmer). Labeled pRNA was incubated with increasing amounts (2.5–160 nM) of recombinant proteins in RNA binding buffer (10 mM Tris–HCl [pH 7.4], 100 mM KCl, 5 mM MgCl_2_) for 30 min on ice and filtered through nitrocellulose membranes (Protran, Whatman). To measure input RNA probes nylon membrane (GE Healthcare) was used. For measuring the specific binding affinity of TIP5/TAM-AT to pRNA (Figure 2d) 40 nM of TIP5/TAM-AT was first incubated with four times excess (2.5–80 nM) of unlabeled MCS-RNA for 30 min on ice. Then, increasing amounts (0.625–20 nM) of [^32^P]-labeled pRNA probe were added and reactions were allowed to stay on ice for additional 1.5 h. After four washes with 10 volumes of RNA binding buffer, retained RNA was monitored by scintillation counting or by PhosphorImaging and quantified with Image Gauge 4.0 quantification software.

### RNA immunoprecipitation assays (RIP)

HEK293T cells were transfected with expression vectors encoding FLAG/HA-tagged wild-type or mutant full-length human TIP5 or a control vector (pCS2+). Forty-eight hours after transfection cells were lysed in 500 μl of RNA immunoprecipitation assays (RIP) lysis buffer (200 mM NaCl, 5 mM MgCl_2_, 10 mM Tris–HCl [pH 7.2], 0.5% NP-40, 1 mM DTT) supplemented with 100 u/ml of RNasin (Promega) and 25 μl/ml of proteinase inhibitor cocktail (Sigma). Lysates were sonicated, spun and digested with 30 u/100 μl of Dnase I (Roche) for 20 min at room temperature. After addition of 5 mM EDTA, FLAG-tagged proteins were bound to M2 beads (Sigma), washed 10× with 10 volumes of RIP lysis buffer. RNA was recovered with Trizol (Invitrogen) and subjected to RT-PCR using the forward primer 5′-CGATGGTGGCGTTTTT (hrDNA/-150/-130F) and the reverse primer 5′-CTCGGAGCGAAAGATATACC (hrDNA/-30/-50R).

## RESULTS

### The TAM domain of TIP5 adopts an extended MBD fold

Sequence alignments of the TAM domain of human TIP5 (BAZ2A) with phylogenetically related homologs (14,47–48) and canonical methyl-CpG binding domains (MBDs) show sequence conservation of the TAM domain in vertebrates, whereas sequence homology with canonical MBDs is less obvious (Figure 1b). Notably, the TAM domain comprises a unique 36 amino acid insert, which is not present in canonical MBDs and has two conserved AT-hooks adjacent to the TAM domain. The phylogenetically conserved region of the TAM domain extends at both the N- and C-terminal ends beyond the borders of the MBD identified by domain predictors, e.g. SMART (49,50) and Pfam (51).

To study the structure and RNA binding properties of the TAM domain, we analyzed TIP5/TAM-AT, a TIP5 fragment comprising residues 496–664, by NMR chemical shifts and ^15^N relaxation experiments (Figure 2a). The ^13^C secondary chemical shifts, ^15^N relaxation rates and low {^1^H}-^15^N heteronuclear NOE values for the amides of residues 496–519 and 622–664 (comprising the AT-hooks) indicate that these regions are flexible and intrinsically disordered, while residues 520–621 are structured and define the globular fold of the TAM domain.

To identify the minimal RNA binding region of TIP5 we performed EMSA using TIP5 versions comprising residues 332–723 (‘TIP5’), 496–664 (‘TIP5/TAM-AT’) and 516–623 (‘TIP5/TAM’) and ^32^P-labeled MCS-RNA. As shown in Figure 2b and c all three TIP5 fragments bind to the RNA. While at 30 nM protein TIP5 quantitatively forms protein–RNA complexes and TIP5/TAM-AT retains 97% of the RNA binding activity, TIP5/TAM has only 75% of the efficiency of TIP5 for RNA binding. These results indicate that the TAM-AT domain comprises the RNA binding region of TIP5 and suggest that the TAM domain is the principal RNA binding domain of TIP5. The presence of super-shifted protein–RNA products formed both by TIP5 and TIP5/TAM-AT, but not by TIP5/TAM, as well as a slight increase in RNA binding affinity observed with TIP5/TAM-AT as compared to TIP5/TAM, suggests that additional auxiliary RNA interaction may be contributed by the AT-hooks. We used FBA to quantitatively determine the binding affinity of TIP5/TAM-AT to pRNA. These experiments show that TIP5/TAM-AT binds to pRNA with a dissociation constant of *K*_D_ = 5.5 nM (Figure 2d).

**Figure 3.**
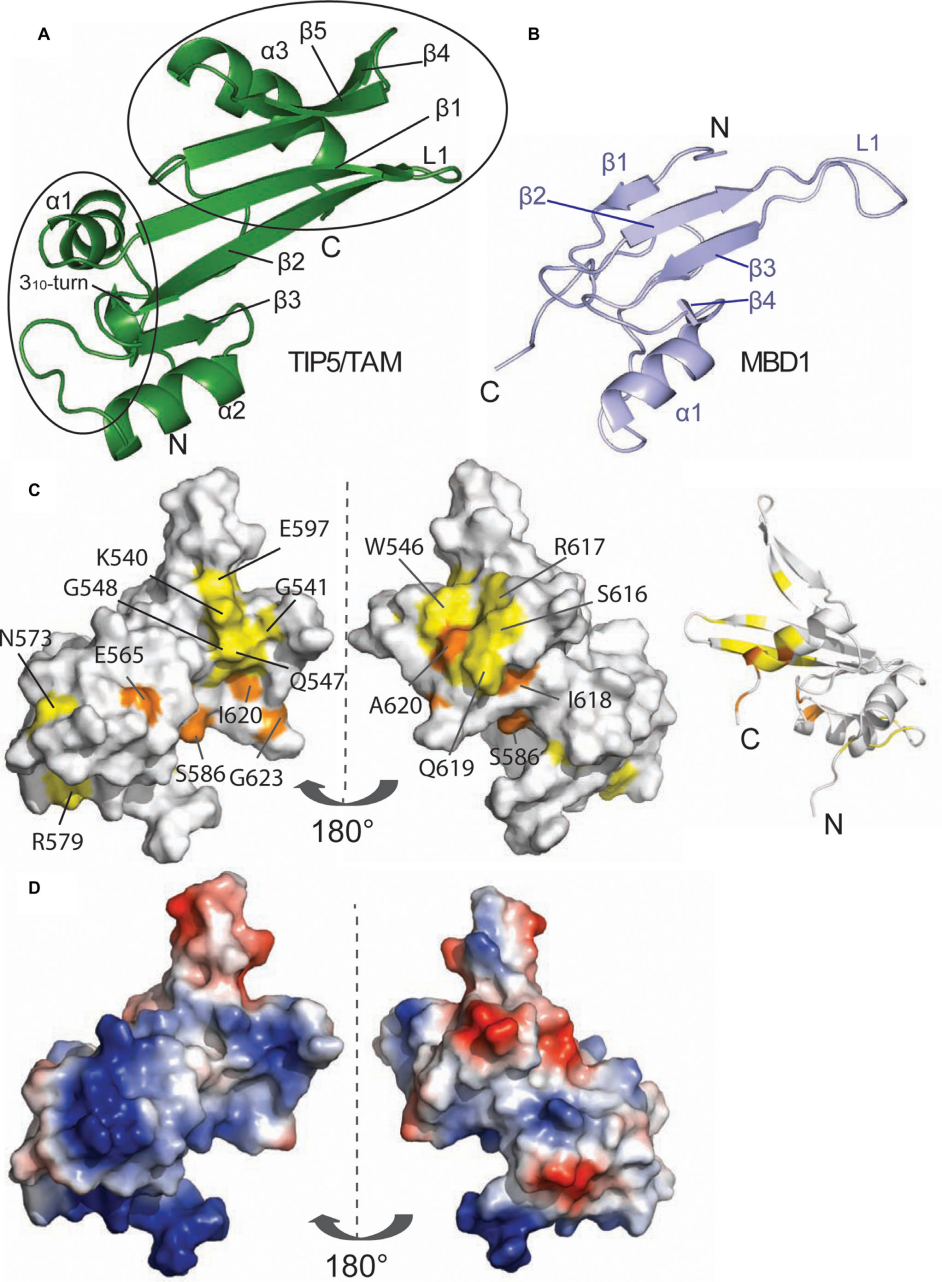
Solution structure of the hTIP5/TAM domain. (**A**) Cartoon representation of the human TIP5/TAM domain. Secondary structure elements and important loops are annotated. The TAM-specific N-terminal helix and the C-terminal α/β motif are highlighted by black circles. (**B**) Cartoon representation of the canonical MBD domain of human MBD1 (light blue, PDB ID: 1D9N) in a comparable view as the TAM domain on the left. Secondary structure elements and the loop L1 that mediates DNA binding of the MBD1 domain are annotated. (**C**) Mapping of the binding surface of TIP5/TAM with pRNA^mini^ based on NMR chemical shift perturbations (Figure 4c) onto a surface representation (left and middle panel) and a ribbon model (right) of the TIP5/TAM domain structure. Left: same view as in (a), middle and right views are rotated by 180°. Colors indicate the RNA binding surface mapped by NMR titrations (Figure 4c and d). Residues with significant chemical shift perturbation are shown in yellow, while residues that exhibit strong intensity reductions upon RNA binding are colored orange as in Figure 4c and d. (**D**) Electrostatic potential of the TAM domain. Vacuum electrostatics surfaces are shown blue for positive and red for negative charges for the TAM domain in the same orientations as in (c).

Next, we determined the solution structure of TIP5/TAM by heteronuclear NMR spectroscopy. The secondary structure of the TAM domain is indicated by ^13^C secondary chemical shifts and supported by H/D exchange experiments (Figure 2a, Supplementary Figure S1a). The ensemble of 20 lowest energy structures is well-defined by the NMR data with a backbone coordinate RMSD of 0.38 ± 0.08 Å and good structural statistics (Supplementary Figure S1b, Table 1). The TAM domain adopts an α/β fold, with a twisted β-sheet comprised of five antiparallel β-strands that pack against three α-helices (Figure 3a). The β-sheet represents a continuous surface, where two short loops connecting β1–β2 and β4–β5 open up like a clamp in the C-terminal region. Helix α2 (Phe563-Arg572) is inserted between the strands β3 and β4 and followed by a 3_10_-helical turn. Helix α3 (Ala611-Ile621) follows the β5 strand at the C-terminus of the protein. The N-terminal helix α1 (Pro522-Gln530) is slightly bent, presumably due to the presence of a conserved proline residue (Pro528). The TAM domain adopts a stable and rigid fold, with only few residues in the linker following helix α2 (Asn573-Arg579), and the β1–β2 with β4–β5 loops showing increased flexibility as indicated by low {^1^H}-^15^N heteronuclear NOE values (Figure 2a; Supplementary Figure S1a). Notably, the linker connecting the 3_10_-helical turn with β4 (Phe583-Gly592) is well-ordered and not flexible.

**Table 1.** Structural statistics for the human TIP5/TAM domain

Number of NMR restraints	
NOE-derived distance restraints^a^	
Total NOE	2732
Intra/inter-residue	607/2125
Sequential (|*i* – *j*| = 1)	738
Medium-range (1 < |*i* – *j*| < 5)	478
Long-range (|*i* – *j*| ≥ 5)	909
Dihedral angle restraints (ϕ/ψ) ^b^	83/85
Residual dipolar couplings^c^	178
Restraint violations (mean and s.d.)	
Distance restraints (Å)	0.013 ± 0.001
Dihedral angle restraints (°)	1.150 ± 0.046
Maximum dihedral angle violation (°)	4.96
Maximum distance constraint violation (Å)	0.28
RDC Q-factor (%)^c^	17.1%
Deviations from idealized geometry	
Bond lengths (Å)	0.004 ± 0.00
Bond angles (°)	0.536 ± 0.01
Impropers (°)	1.232 ± 0.05
Coordinate r.m.s. deviation (Å)	
Heavy	0.98 ±0.13
Backbone	0.32± 0.07

^a^Distance restraints were employed with a soft square well potential using an energy constant of 50kcal mol^−1^ Å^−2^.

^b^Torsion angle restraints derived from TALOS_+_ (31) were applied to φ, ψ backbone angles using energy constants of 200 kcal mol^−1^rad^−2^.

^c^Residual dipolar couplings (RDCs) were employed with a harmonic potential using an energy constant of 0.8 kcal mol^−1^Hz^−2^ for the ^1^H^N^-^15^N RDCs and 0.4 kcal mol^−1^Hz^−2^ for the ^13^C’-^15^N RDCs. Q-factor as defined by (46).

Structural statistics are given for the 20 lowest-energy structures after water refinement out of 200 calculated for residues 516–624 of the human TIP5/TAM domain. Pair wise coordinate r.m.s. deviations are calculated for residues 522–621. The CNS E_repel_ function was used to simulate van der Waals interactions with an energy constant of 25kcal mol^−1^ Å^−4^ using PROLSQ van der Waals radii (41). Backbone torsion angles for 92.1, 7.8 and 0.1% of all residues are found in the core, allowed and generally allowed regions of the Ramachandran plot, none in disallowed regions.

Although the structural core of the TAM domain resembles the MBD domain (Figure 3b) it exhibits unique N- and C-terminal extensions. Compared to the MBD fold the TAM domain has an additional N-terminal helix (α1), a small α/β motif comprising β4, β5 and a helix α3 at the C-terminus. The β1–β2 loop in the TAM domain corresponds to the L1 loop in MBDs which normally mediates contacts with the DNA backbone (52–54). However, the β1–β2 loop in the TAM domain is much shorter and adopts a different conformation, suggesting that this specific structural element may not contact methylated DNA and has a distinct role. Structure-based sequence alignments show that the unique structural elements of the TAM domain, i.e. specifically the small α/β motif, correspond to unique sequence insertions (Figure 1b) that are not present in canonical MBD domains.

### Unique structural elements mediate the interaction of TIP5/TAM with RNA

**Figure 4.**
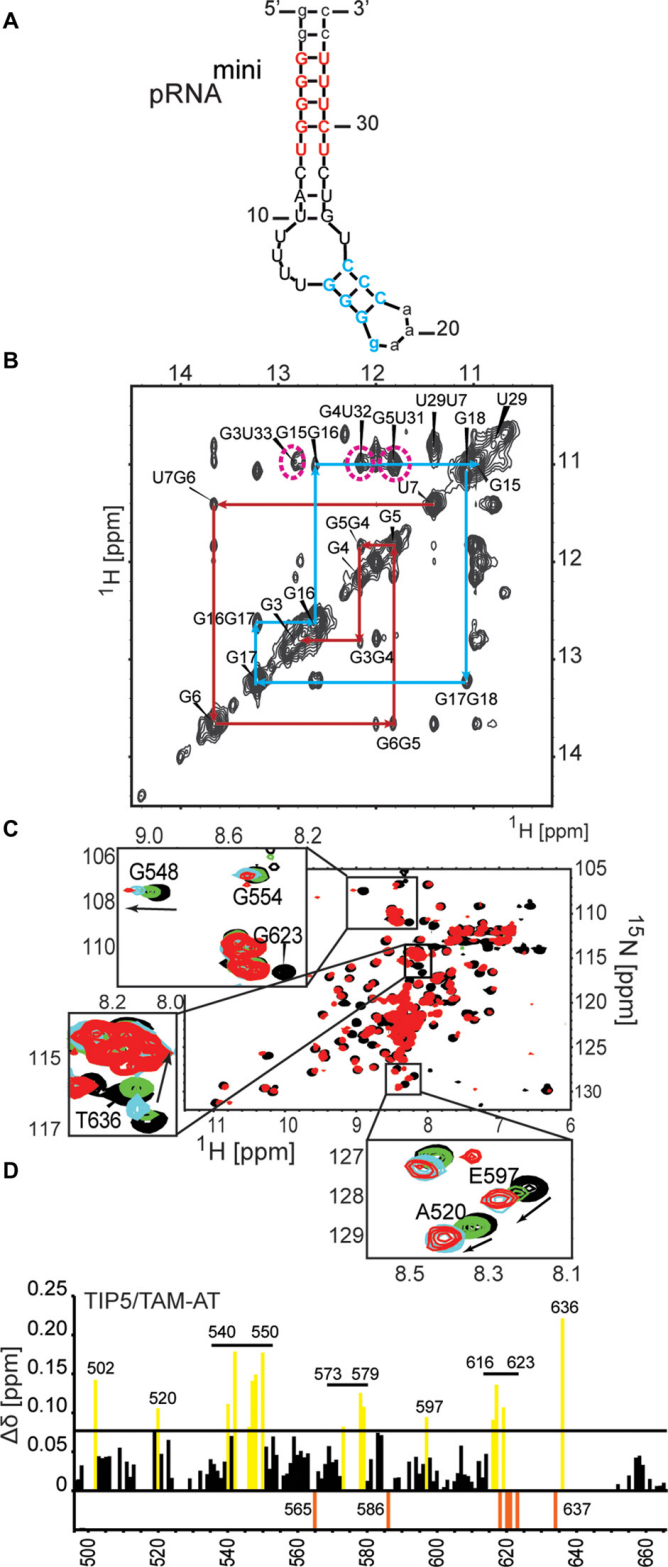
pRNA binding of the TAM domain. (**A**) pRNA^mini^ preserves the structural features of the pRNA stem recognized by TIP5. The secondary structure was calculated by Mfold (55). Nucleotides added for stabilization of the construct are shown in lower-case letters. (**B**) Confirmation of the secondary structure of pRNA^mini^ by an imino NOESY NMR spectrum. The sequential connectivities between imino protons of neighboring base pairs in the two stem regions are indicated by red and blue lines, respectively, matching the colors indicated for the RNA stem in (a). The characteristic imino signals of the G-U and U-U base pairs have chemical shifts around 11–12 ppm as expected. G-U cross peaks are indicated by magenta dotted lines. The G3-U33 cross peak is broadened possibly due to its terminal position in the pRNA^mini^ stem. (**C**) Superposition of ^1^H,^15^N HSQC NMR spectra of ^15^N-labeled, 50% random fractional deuterated TIP5/TAM-AT free (black) and in the presence of a 1.2 molar excess of pRNA^mini^ (red). Insets show close-up views for specific residues upon addition of 0.2 (green), 0.5 (blue) and 1.2 (red) molar equivalents of pRNA^mini^. Arrows indicate the direction of the peak shift. (**D**) NMR chemical shift perturbations observed for backbone amides in TIP5/TAM-AT at 1.2 molar excess of pRNA. NMR signals that experience line-broadening upon titration of the RNA are shown as orange negative bars. Residues with chemical shift changes larger than one standard deviation from the average (Δδ = 0.078 ppm) are colored yellow.

To map the RNA binding surface of the TIP5/TAM-AT domain by NMR chemical shift perturbation experiments, we designed a shorter version of pRNA, dubbed pRNA^mini^. pRNA^mini^ has been previously described and represents a fusion of pRNA nucleotides -127/-113 with nucleotides -60/-49. This preserves a specific stem-loop structure that is recognized by TIP5 with features conserved between mouse and human (10,55) (Figure 4a, Supplementary Figure S2). The secondary structure and base pairing of pRNA^mini^ was confirmed by an imino NOESY spectrum (Figure 4b). In competition EMSA experiments we found that pRNA and pRNA^mini^ are able to compete binding of non-specific MCS RNA to TIP5 at low and high nanomolar concentrations, respectively (Supplementary Figure S2). This is consistent with the low nanomolar affinity observed for the pRNA-TIP5 interaction in EMSA experiments (Figure 2d). It also indicates that pRNA^mini^ binds to TIP5 with a (high) nanomolar dissociation constant. Thus, although pRNA^mini^ has (about 10-fold) reduced affinity to TIP5, our data suggest that it harbors important structural features required for efficient binding to TIP5 and was therefore used for NMR titration experiments.

We monitored binding of TIP5/TAM-AT to pRNA^mini^ in a series of ^1^H,^15^N NMR correlation spectra with ^15^N labeled, 50% deuterated TIP5/TAM-AT protein at increasing molar equivalents of RNA. Numerous NMR signals experience large chemical shift changes and/or line-broadening upon addition of pRNA^mini^ (Figure 4c and d). The binding kinetics is in the fast to intermediate exchange regime with respect to the NMR chemical shift timescale, consistent with a dissociation constant in a high nanomolar range (56) as indicated in the competition EMSA experiments (Supplementary Figure S2). Interestingly, binding is saturated at 1:0.5 molar ratio of protein to RNA, suggesting that the RNA has two binding sites for TIP5/TAM-AT. The RNA binding surface of the TAM domain maps to the small α/β motif comprising β4, β5 and helix α3, i.e. the region that is unique to the TAM domain (Figure 3c). This RNA binding surface is flanked by a positively charged region, indicating that electrostatic interactions contribute to RNA binding. (Figure 3d). The AT-hooks harbor a number of additional positively charged residues. In the NMR titrations, many residues of the AT-hooks are strongly affected by RNA addition, although the chemical shift changes could in most cases not be traced to quantitatively analyze the chemical shift perturbations. Nevertheless, this indicates that the AT-hooks also contribute to the RNA binding, presumably by mediating additional electrostatic contacts with the RNA. In the free protein the AT-hooks are intrinsically disordered (Figure 2a), while in the RNA complex they may become more ordered by forming charged interactions with the RNA. This is supported by line broadening and/or large chemical shift changes for the few residues that could be analyzed in the NMR titration (i.e. A634, T636; Figure 4c and d). Together, the TAM domain harbors a novel RNA binding surface that involves a unique C-terminal α/β motif that is absent in canonical MBDs, thus rationalizing the unique capability of TAM to bind RNA. The AT-hooks likely contribute additional non-specific electrostatic contacts with the RNA.

We also monitored the interaction of TIP5/TAM with other RNAs (Supplementary Figure S3). While negligible chemical shift changes were observed with single-stranded poly-U RNA, significant spectral changes occurred in the presence of an RNA hairpin found in the A-repeat region of Xist a noncoding RNA that is required for X-chromosome inactivation (57,58). Interestingly, the TAM residues affected in NMR titrations upon binding to pRNA^mini^ or Xist RNA are similar (Supplementary Figure S3c). This observation is consistent with the EMSA data and previous studies showing that TIP5 does not recognize a specific RNA sequence but rather distinct structural features, e.g. double-helical regions in stem-loop structures that may also be present in other RNAs.

### Mutational analysis of the TAM–RNA interface

**Figure 5.**
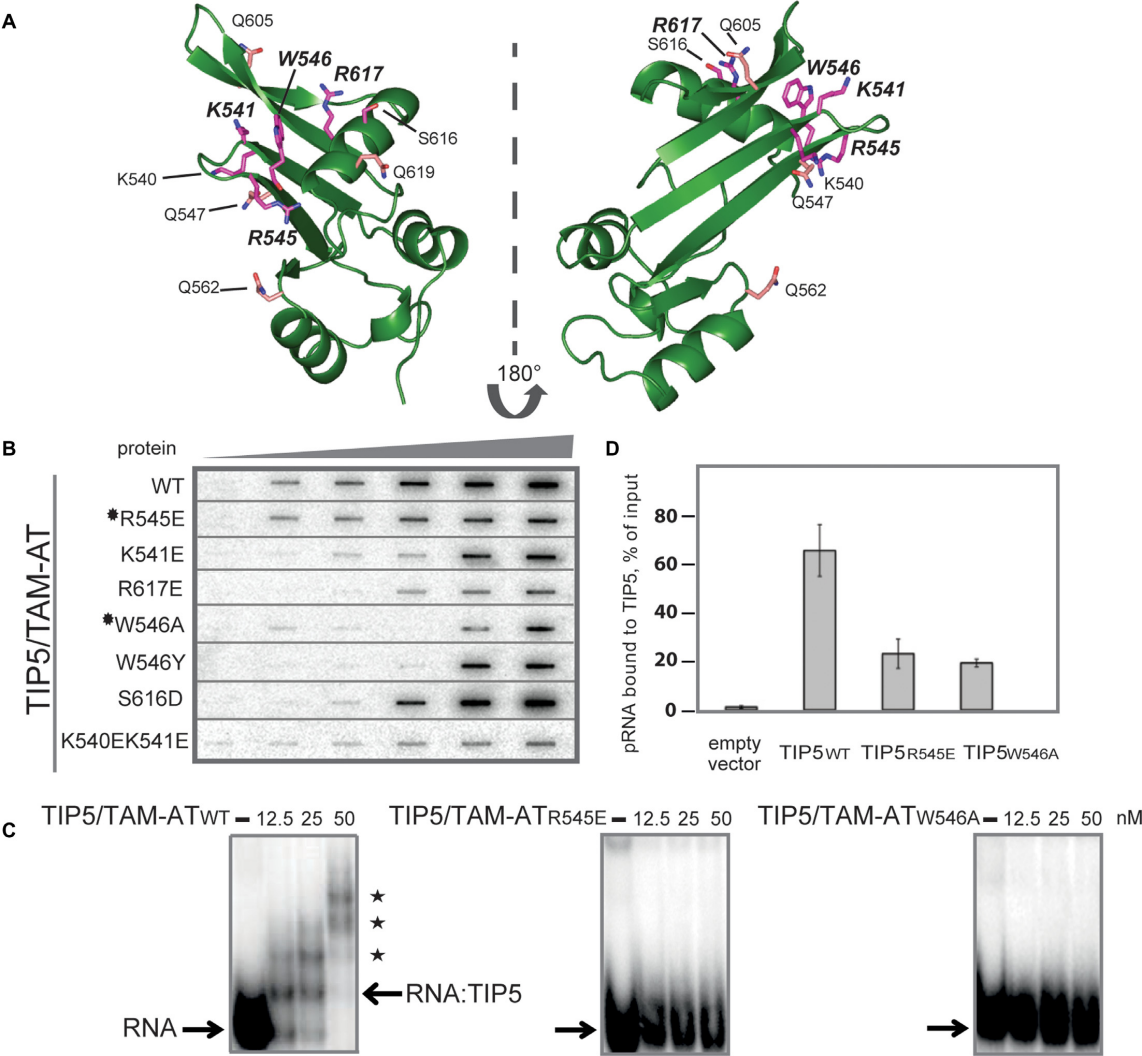
Mutational analysis of TAM/pRNA interaction. (**A**) Side chains of residues probed by mutational analysis are shown in magenta on the structure of the TIP5/TAM domain. Residues which are involved in RNA binding are annotated with larger font. Side chains shown in pink are controls, not expected to be involved in RNA binding. (**B**) RNA binding of wild-type and mutant TIP5/TAM-AT. The triangle on top indicates increasing protein concentrations (0, 5, 10, 20, 40 and 80 nM) of wild-type TIP5/TAM-AT (WT) or the indicated TIP5/TAM-AT mutants, which were incubated with 10 nM of [^32^P]-pRNA. Protein–RNA complexes were retained on nitrocellulose filters and visualized by phosphorimaging. Mutants selected for further analyses (R545E and W546A) are marked with black stars. (**C**) EMSA experiments monitoring binding of wild-type TIP5/TAM-AT and mutants R545E and W546A to RNA. 12.5, 25, 50 nM of the respective proteins were incubated with 2.5 nM of [^32^P]-labeled RNA. Positions of free RNA and RNA:TIP5 complexes are indicated by arrows. Supershifts indicating higher order complexes of TIP5 binding with several RNA molecules are marked with asterisks. (**D**) RNA immunoprecipitation experiments monitoring binding of wild-type and mutant TIP5 to pRNA *in vivo*. HEK293T cells were transfected with expression vectors encoding FLAG-tagged wild-type TIP5, TIP5/R545E or TIP5/W546A. TIP5 was immunoprecipitated with anti-FLAG antibodies and associated pRNA was monitored by RT-qPCR.

To validate the NMR data and examine the functional significance of the RNA binding surface, we monitored RNA binding of a panel of mutants that comprise specific amino acid exchanges in the TAM domain. Solvent exposed amino acid residues within the pRNA binding interface as well as conserved residues within the β-sheet surface of MBD domains (52) were altered, yielding the TAM variants K541E, K540E/K541E, R545E, W546Y, W546A, Q547A, Q562A, Q605A, S616D, R617E and Q619A (Figure 5a). The structural integrity of all proteins was monitored by NMR (Supplementary Figure S4). We also investigated the W551/Y552 double mutation in human TIP5, as we have previously shown that the corresponding mouse TIP5 W531G/Y532A mutant abolishes pRNA binding and strongly affects heterochromatic hallmarks when overexpressed in eukaryotic cells (6). The corresponding residues in human TIP5 (W551 and Y552) contribute to the hydrophobic core of the TAM domain fold. Consistent with this the double mutation W551G/Y552A is structurally unstable as the recombinant TAM W551G/Y552A double mutant is partially unfolded (Supplementary Figure S5). This shows that the structural integrity of the TAM domain is required for pRNA binding and functional activity of TIP5.

Next, we monitored RNA binding of TIP5, TIP5/TAM-AT and several TIP5/TAM-AT mutants using filter binding assays and EMSA. As expected, mutations altering residues remote from the TAM-pRNA interface (Q547A, Q562A, Q605A, Q619A and S616D) did not significantly affect RNA binding without affecting the tertiary structure (Supplementary Figure S4 and data not shown). In contrast, mutations within the RNA binding interface (K541E, W546Y, W546A and R617E) significantly reduced binding to pRNA, without affecting the domain fold (Figure 5b, Supplementary Figure 4). NMR spectra data indicate that the TAM domain fold is destabilized in the K540E/K541E double mutant. Thus, impaired RNA binding of this mutant is likely due to misfolding and/or aggregation of the TAM domain, underlining the importance of structural integrity for RNA binding of TIP5/TAM. Consistent with the filter binding experiments, electrophoretic shift mobility assays revealed that RNA binding of mutants R545E and W546A was strongly decreased (Figure 5c), indicating that R545 and W546 are directly involved in the interaction of TIP5 with RNA.

To examine whether mutations that impair pRNA binding *in vitro* also affect TIP5-pRNA interaction *in vivo*, we performed RIP assays to monitor the association of pRNA with wild-type and mutant TIP5. Lysates from cells expressing FLAG-tagged TIP5, TIP5-R545E or TIP5-W546A were precipitated with anti-FLAG antibodies, and co-precipitated pRNA was monitored by RT-PCR. Cells transfected with the empty vector were used as a control. As shown in Figure 5d, RNA binding of the R545E or W546A mutant was markedly decreased as compared to wildtype TIP5. These results are in accord with the *in vitro* data demonstrating the functional relevance of residues R545 and W546 for TIP5 binding to RNA.

## DISCUSSION

We report the solution structure of the TAM (TIP5/ARBP/MBD) domain of human TIP5 and show that it represents a novel extended MBD fold that is normally found in MBD-containing proteins such as MeCP1, MBD1, MBD2, MBD3 and MBD4. MBD-containing proteins function by recruiting various protein complexes to methyl-CpG sites, linking DNA methylation with chromatin modification and transcriptional repression. Significantly, the structurally related TAM domain does not exhibit binding preference to methylated DNA but associates with RNA. Previous studies have shown that the interaction of TIP5 with pRNA is crucial for targeting the chromatin remodeling complex NoRC to nucleoli and the establishment of heterochromatic features at a subset of rRNA genes (6). TIP5 binds to a phylogenetically conserved hairpin structure of pRNA, which is required for epigenetic regulation of rRNA genes (10).

Our structure reveals that the structural core of the TAM domain resembles the MBDs of MBD1 and MeCP2. However, the TAM domain comprises unique C-terminal extensions, with the C-terminal α/β motif not being present in canonical MBD structures. Significantly, the positively charged C-terminal α/β motif constitutes the binding interface of TIP5 and pRNA. The sequence of the TAM domain and residues involved in RNA binding are conserved in vertebrates, underscoring the functional importance of the TAM domain distinct from MBD proteins. Mutational analysis revealed that residues in the RNA binding surface are required for RNA binding *in vitro* and *in vivo*. We found that two of the tested residues, W546 and R545, are the most important for RNA binding. Notably, these residues are not conserved in human MBDs, suggesting that the interaction of TAM-containing proteins with specific regulatory RNA may guide chromatin modifying enzymes to regulatory gene regions.

**Figure 6.**
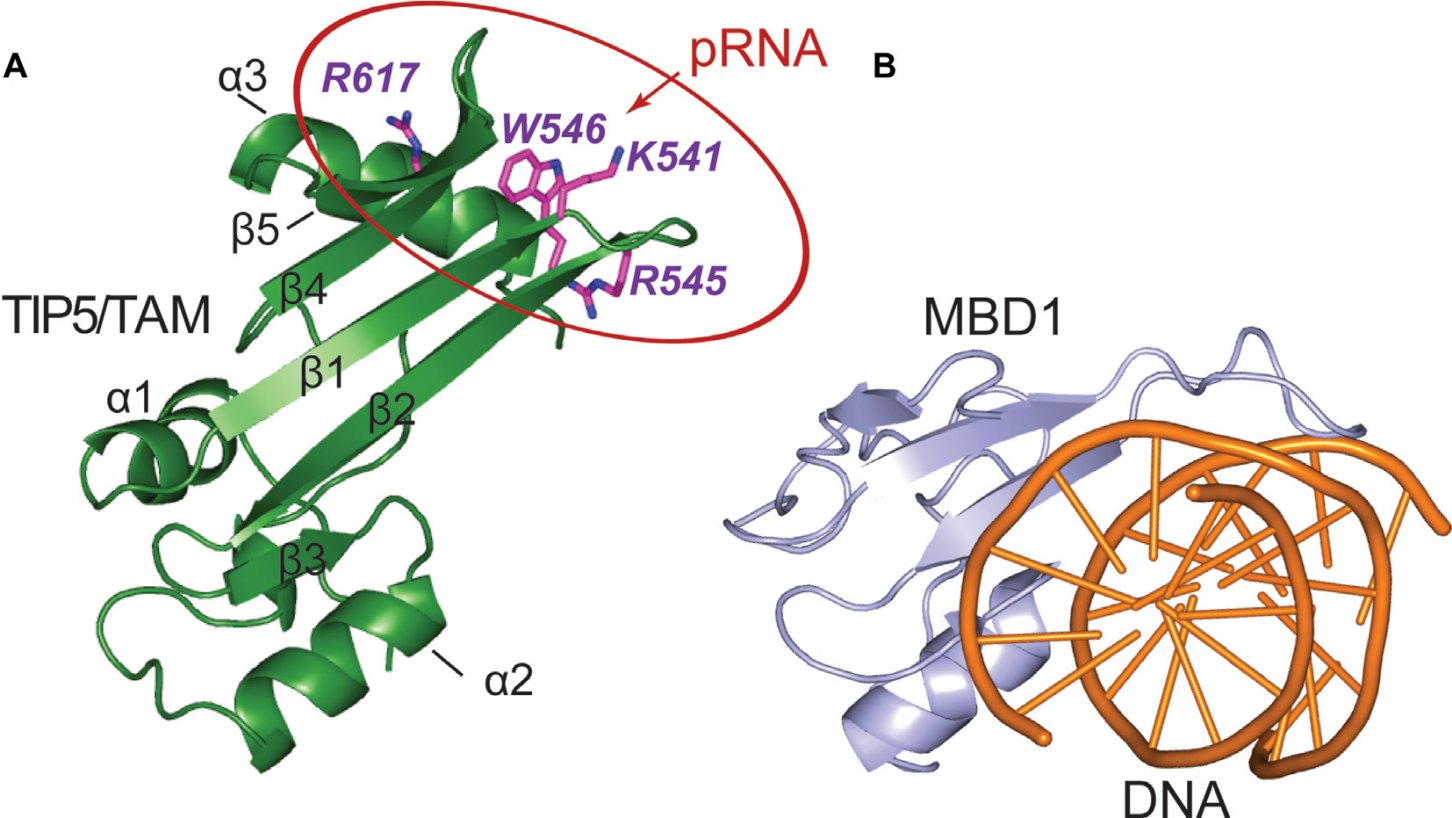
Comparison of TAM and MBD nucleic acid binding domains. (**A**) The pRNA binding surface of the TIP5/TAM domain. Side chains of residues important for RNA binding are shown on a cartoon representation of the TIP5/TAM domain structure. (**B**) Cartoon representation of the canonical MBD domain from MBD1 (light blue) in complex with methylated DNA shown as orange cartoon (PDB ID: 1IG4) (52).

Canonical MBDs recognize methylated CpGs in DNA by hydrophobic residues located at the β-sheet surface, with additional DNA backbone contacts mediated by the extended loop L1 (52) (Figure 6). Although the fold of the TAM domain resembles the MBD domain, TAM does not exhibit binding preference for methylated DNA (Supplementary Figure S6). This may be explained by the fact that amino acids that mediate specific recognition of methylated CpG regions by the MBD domain are not conserved in the TAM domain (Figure 1b). Hence, the TIP5/TAM domain has evolved as a specific pRNA recognition domain that is required for NoRC recruitment to the rDNA promoter.

Interestingly, our NMR titrations show that the TAM domain also binds to a hairpin found in Xist RNA, a non-coding RNA involved in dosage compensation and X-chromosome inactivation. A common structural feature between pRNA and Xist RNA are double-helical regions. This suggests that the TIP5/TAM domain preferably recognizes double-helical regions in NoRC-associated RNAs. This may explain why our NMR titrations indicate that two TAM-AT proteins can bind to pRNA^mini^, which has two helical stem regions. Also, the observation of supershifts in the EMSA experiments demonstrates the formation of higher order complexes, which could involve binding of TIP5 to different helical stem regions in larger RNA ligands.

A general preference for helical regions in RNAs by TIP5 is supported by recent data showing that the function of NoRC is not restricted to silencing of rRNA genes but is also required for heterochromatin formation and silencing of centromeres and telomeres (12). Like rRNA genes, centromeres and telomeres are organized in repeated clusters and exhibit a repressive heterochromatic structure. This heterochromatic structure depends on specific ncRNAs, i.e. satellite transcripts originating from the centromeric core region and on TERRA (‘telomeric repeat-containing RNA’), respectively. Indeed, TERRA was shown to be associated with TIP5, supporting that TERRA serves a guiding function in NoRC-dependent heterochromatin formation at telomeres (12). It is tempting to speculate that the interaction of NoRC with pRNA, satellite RNA and TERRA relies on the recognition of double-helical regions in these RNAs which is recognized by the TAM domain of TIP5. Future structural characterization of full-length TIP5 and its interaction with different RNAs will give further insights into the versatility of RNA recognition motifs and their involvement in diverse biological functions.

## ACCESSION NUMBERS

The atomic coordinates for the NMR ensemble of the TIP5/TAM domain have been deposited in the Protein Data Bank under accession number 5AGQ. The chemical shift assignments have been deposited in the Biological Magnetic Resonance Data Bank under accession number 26517.

## SUPPLEMENTARY DATA

Supplementary Data are available at NAR Online.

SUPPLEMENTARY DATA

## References

[1] McStay B., Grummt I. (2008). The epigenetics of rRNA genes: from molecular to chromosome biology. Annu. Rev. Cell Dev. Biol..

[2] Strohner R., Nemeth A., Jansa P., Hofmann-Rohrer U., Santoro R., Langst G., Grummt I. (2001). NoRC–a novel member of mammalian ISWI-containing chromatin remodeling machines. Embo J..

[3] Santoro R., Li J., Grummt I. (2002). The nucleolar remodeling complex NoRC mediates heterochromatin formation and silencing of ribosomal gene transcription. Nat. Genet..

[4] Zhou Y., Santoro R., Grummt I. (2002). The chromatin remodeling complex NoRC targets HDAC1 to the ribosomal gene promoter and represses RNA polymerase I transcription. EMBO J..

[5] Li J., Langst G., Grummt I. (2006). NoRC-dependent nucleosome positioning silences rRNA genes. EMBO J..

[6] Mayer C., Schmitz K.M., Li J., Grummt I., Santoro R. (2006). Intergenic transcripts regulate the epigenetic state of rRNA genes. Mol. Cell.

[7] Schmitz K.M., Mayer C., Postepska A., Grummt I. (2010). Interaction of noncoding RNA with the rDNA promoter mediates recruitment of DNMT3b and silencing of rRNA genes. Genes Dev..

[8] Zhou Y., Grummt I. (2005). The PHD finger/bromodomain of NoRC interacts with acetylated histone H4K16 and is sufficient for rDNA silencing. Curr. Biol..

[9] Santoro R., Schmitz K.M., Sandoval J., Grummt I. (2010). Intergenic transcripts originating from a subclass of ribosomal DNA repeats silence ribosomal RNA genes in trans. EMBO Rep..

[10] Mayer C., Neubert M., Grummt I. (2008). The structure of NoRC-associated RNA is crucial for targeting the chromatin remodelling complex NoRC to the nucleolus. EMBO Rep..

[11] Savic N., Bar D., Leone S., Frommel S.C., Weber F.A., Vollenweider E., Ferrari E., Ziegler U., Kaech A., Shakhova O. (2014). lncRNA maturation to initiate heterochromatin formation in the nucleolus is required for exit from pluripotency in ESCs. Cell Stem Cell.

[12] Postepska-Igielska A., Krunic D., Schmitt N., Greulich-Bode K.M., Boukamp P., Grummt I. (2013). The chromatin remodelling complex NoRC safeguards genome stability by heterochromatin formation at telomeres and centromeres. EMBO Rep..

[13] Gu L., Frommel S.C., Oakes C.C., Simon R., Grupp K., Gerig C.Y., Bar D., Robinson M.D., Baer C., Weiss M. (2015). BAZ2A (TIP5) is involved in epigenetic alterations in prostate cancer and its overexpression predicts disease recurrence. Nat. Genet..

[14] Hendrich B., Tweedie S. (2003). The methyl-CpG binding domain and the evolving role of DNA methylation in animals. Trends Genet..

[15] Aravind L., Landsman D. (1998). AT-hook motifs identified in a wide variety of DNA-binding proteins. Nucleic Acids Res..

[16] Jeffery L., Nakielny S. (2004). Components of the DNA methylation system of chromatin control are RNA-binding proteins. J. Biol. Chem..

[17] Lukavsky P.J., Puglisi J.D. (2004). Large-scale preparation and purification of polyacrylamide-free RNA oligonucleotides. RNA.

[18] Wyatt J.R., Chastain M., Puglisi J.D. (1991). Synthesis and purification of large amounts of RNA oligonucleotides. Biotechniques.

[19] Kao C., Zheng M., Rudisser S. (1999). A simple and efficient method to reduce nontemplated nucleotide addition at the 3 terminus of RNAs transcribed by T7 RNA polymerase. RNA.

[20] Delaglio F., Grzesiek S., Vuister G.W., Zhu G., Pfeifer J., Bax A. (1995). NMRPipe: a multidimensional spectral processing system based on UNIX pipes. J. Biomol. NMR.

[21] Sattler M., Schleucher J., Griesinger C. (1999). Heteronuclear multidimensional NMR experiments for the structure determination of proteins in solution employing pulsed field gradients. Prog. Nucl. Magn. Reson. Spectrosc..

[22] Pastore A., Saudek V. (1990). The relationship between chemical shift and secondary structure in proteins. J. Magn. Reson..

[23] Spera S., Bax A. (1991). Empirical correlation between protein backbone conformation and C.alpha. and C.beta. 13C nuclear magnetic resonance chemical shifts. J. Am. Chem. Soc..

[24] Yamazaki T., Forman-Kay J.D., Kay L.E. (1993). Two-dimensional NMR experiments for correlating carbon-13.beta. and proton.delta./.epsilon. chemical shifts of aromatic residues in 13C-labeled proteins via scalar couplings. J. Am. Chem. Soc..

[25] Pelton J.G., Torchia D.A., Meadow N.D., Roseman S. (1993). Tautomeric states of the active-site histidines of phosphorylated and unphosphorylated IIIGlc, a signal-transducing protein from Escherichia coli, using two-dimensional heteronuclear NMR techniques. Protein Sci..

[26] Senn H., Werner B., Messerle B.A., Weber C., Traber R., Wuethrich K. (1989). Stereospecific assignment of the methyl 1H NMR lines of valine and leucine in polypeptides by nonrandom 13C labelling. FEBS Lett..

[27] Neri D., Szyperski T., Otting G., Senn H., Wüthrich K. (1989). Stereospecific nuclear magnetic resonance assignments of the methyl groups of valine and leucine in the DNA-binding domain of the 434 repressor by biosynthetically directed fractional ^13^C labeling. Biochemistry.

[28] Jung Y.S., Zweckstetter M. (2004). Mars—robust automatic backbone assignment of proteins. J. Biomol. NMR.

[29] Guntert P. (2004). Automated NMR structure calculation with CYANA. Methods Mol. Biol..

[30] Shen Y., Delaglio F., Cornilescu G., Bax A. (2009). TALOS+: a hybrid method for predicting protein backbone torsion angles from NMR chemical shifts. J. Biomol. NMR.

[31] Cordier F., Dingley A.J., Grzesiek S. (1999). A doublet-separated sensitivity-enhanced HSQC for the determination of scalar and dipolar one-bond J-couplings. J. Biomol. NMR.

[32] Ishii Y., Markus M.A., Tycko R. (2001). Controlling residual dipolar couplings in high-resolution NMR of proteins by strain induced alignment in a gel. J. Biomol. NMR.

[33] Ottiger M., Bax A. (1999). Bicelle-based liquid crystals for NMR-measurement of dipolar couplings at acidic and basic pH values. J. Biomol. NMR.

[34] Farrow N.A., Muhandiram R., Singer A.U., Pascal S.M., Kay C.M., Gish G., Shoelson S.E., Pawson T., Forman-Kay J.D., Kay L.E. (1994). Backbone dynamics of a free and phosphopeptide-complexed Src homology 2 domain studied by 15N NMR relaxation. Biochemistry.

[35] Akke M., Palmer A.G. (1996). Monitoring macromolecular motions on microsecond to millisecond time scales by R1ρ−R1 constant relaxation time NMR spectroscopy. J. Am. Chem. Soc..

[36] Kay L.E., Torchia D.A., Bax A. (1989). Backbone dynamics of proteins as studied by 15N inverse detected heteronuclear NMR spectroscopy: application to staphylococcal nuclease. Biochemistry.

[37] Bruschweiler R., Liao X., Wright P. (1995). Long-range motional restrictions in a multidomain zinc-finger protein from anisotropic tumbling. Science.

[38] Daragan V.A., Mayo K.H. (1997). Motional model analyses of protein and peptide dynamics using 13C and 15N NMR relaxation. Prog. Nucl. Magn. Reson. Spectrosc..

[39] Schanda P., Brutscher B. (2005). Very fast two-dimensional NMR spectroscopy for real-time investigation of dynamic events in proteins on the time scale of seconds. J. Am. Chem. Soc..

[40] Linge J.P., Habeck M., Rieping W., Nilges M. (2003). ARIA: automated NOE assignment and NMR structure calculation. Bioinformatics.

[41] Linge J.P., Williams M.A., Spronk C.A., Bonvin A.M., Nilges M. (2003). Refinement of protein structures in explicit solvent. Proteins.

[42] Doreleijers J.F., Sousa da Silva A.W., Krieger E., Nabuurs S.B., Spronk C.A., Stevens T.J., Vranken W.F., Vriend G., Vuister G.W. (2012). CING: an integrated residue-based structure validation program suite. J. Biomol. NMR.

[43] Laskowski R.A., Macarthur M.W., Moss D.S., Thornton J.M. (1993). PROCHECK: a program to check the stereochemical quality of protein structures. J. Appl. Cryst..

[44] Vriend G., Sander C. (1993). Quality control of protein models: directional atomic contact analysis. J. Appl. Crystallogr..

[45] Cornilescu G., Marquardt J.L., Ottiger M., Bax A. (1998). Validation of protein structure from anisotropic carbonyl chemical shifts in a dilute liquid crystalline phase. J. Am. Chem. Soc..

[46] Stepanov A.S., Voronina A.S., Ovchinnikov L.P., Spirin A.S. (1971). RNA-binding protein factor of animal cell extracts. FEBS Lett..

[47] Edgar R.C. (2004). MUSCLE: multiple sequence alignment with high accuracy and high throughput. Nucleic Acids Res..

[48] Larkin M.A., Blackshields G., Brown N.P., Chenna R., McGettigan P.A., McWilliam H., Valentin F., Wallace I.M., Wilm A., Lopez R. (2007). Clustal W and Clustal X version 2.0. Bioinformatics.

[49] Letunic I., Doerks T., Bork P. (2009). SMART 6: recent updates and new developments. Nucleic Acids Res..

[50] Schultz J., Milpetz F., Bork P., Ponting C.P. (1998). SMART, a simple modular architecture research tool: identification of signaling domains. Proc. Natl. Acad. Sci. U.S.A..

[51] Finn R.D., Mistry J., Tate J., Coggill P., Heger A., Pollington J.E., Gavin O.L., Gunasekaran P., Ceric G., Forslund K. (2010). The Pfam protein families database. Nucleic Acids Res..

[52] Ohki I., Shimotake N., Fujita N., Jee J., Ikegami T., Nakao M., Shirakawa M. (2001). Solution structure of the methyl-CpG binding domain of human MBD1 in complex with methylated DNA. Cell.

[53] Ho K.L., McNae I.W., Schmiedeberg L., Klose R.J., Bird A.P., Walkinshaw M.D. (2008). MeCP2 binding to DNA depends upon hydration at methyl-CpG. Mol. Cell.

[54] Scarsdale J.N., Webb H.D., Ginder G.D., Williams D.C. Jr (2011). Solution structure and dynamic analysis of chicken MBD2 methyl binding domain bound to a target-methylated DNA sequence. Nucleic Acids Res..

[55] Zuker M. (2003). Mfold web server for nucleic acid folding and hybridization prediction. Nucleic Acids Res..

[56] Gobl C., Madl T., Simon B., Sattler M. (2014). NMR approaches for structural analysis of multidomain proteins and complexes in solution. Prog. Nucl. Magn. Reson. Spectrosc..

[57] Postepska-Igielska A., Grummt I. (2014). NoRC silences rRNA genes, telomeres, and centromeres. Cell Cycle.

[58] Bierhoff H., Postepska-Igielska A., Grummt I. (2014). Noisy silence: non-coding RNA and heterochromatin formation at repetitive elements. Epigenetics.

