# Supplementary material for: A novel RNA binding surface of the TAM domain of TIP5/BAZ2A mediates epigenetic regulation of rRNA genes
Source: Nucleic Acids Res. 2015 Apr 27;43(10):5208–20. doi: 10.1093/nar/gkv365 (PMC4446428; doi:10.1093/nar/gkv365)
Supplement: SUPPLEMENTARY DATA [file supp_43_10_5208__index.html]

A novel RNA binding surface of the TAM domain of TIP5/BAZ2A mediates epigenetic regulation of rRNA genes — A novel RNA binding surface of the TAM domain of TIP5/BAZ2A mediates epigenetic regulation of rRNA genes — SUPPLEMENTARY DATA 

# A novel RNA binding surface of the TAM domain of TIP5/BAZ2A mediates epigenetic regulation of rRNA genes

## SUPPLEMENTARY DATA

**Files in this Data Supplement:**

- SUPPLEMENTARY DATA
