# Supplementary material for: A novel RNA binding surface of the TAM domain of TIP5/BAZ2A mediates epigenetic regulation of rRNA genes
Source: Nucleic Acids Res. 2015 Apr 27;43(10):5208–20. doi: 10.1093/nar/gkv365 (PMC4446428; doi:10.1093/nar/gkv365)
Supplement: SUPPLEMENTARY DATA [file supp_gkv365_nar-03366-m-2014-File009.pdf]

SUPPLEMENTARY INFORMATION

Supplementary Fig. 1

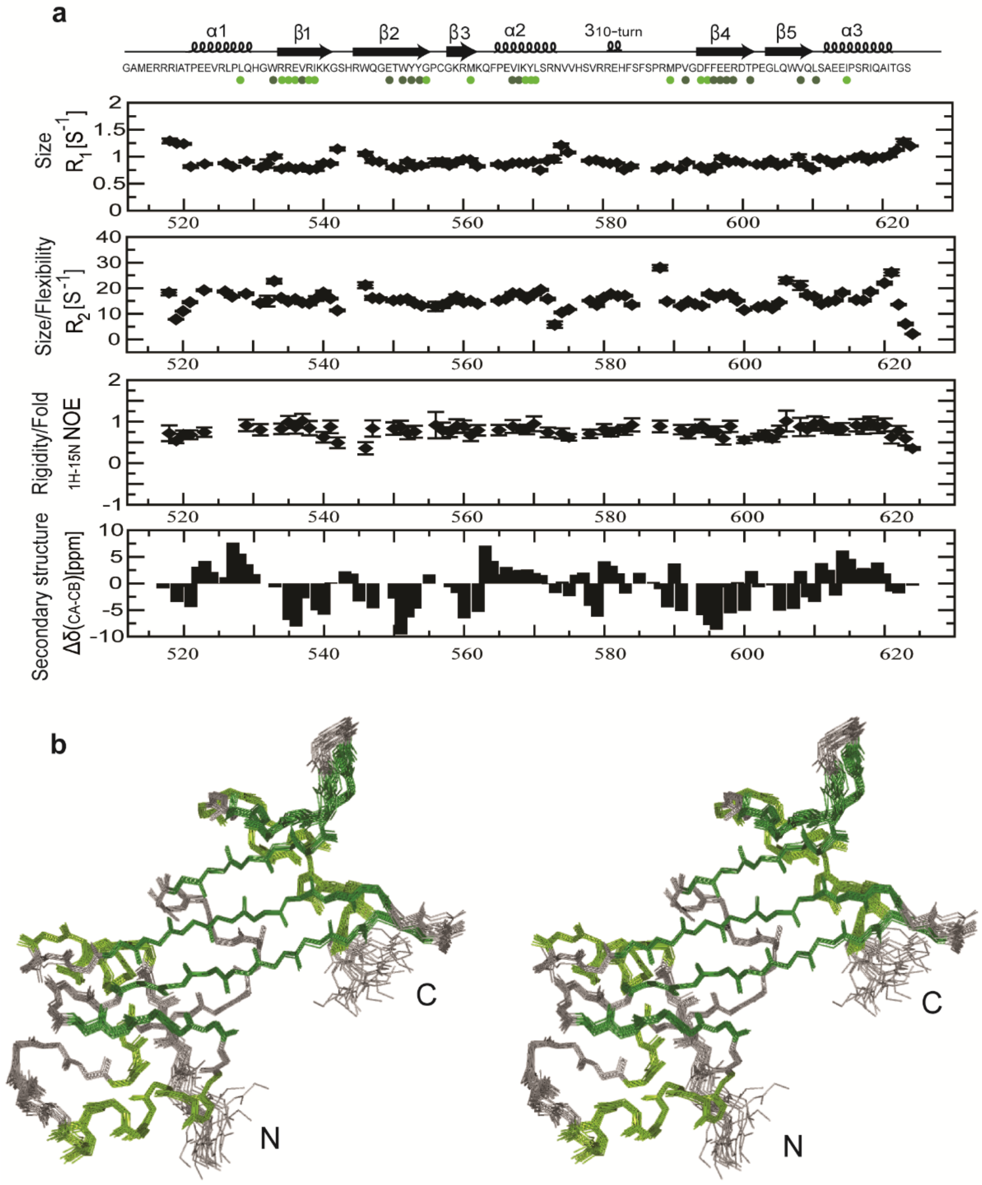

Suppl. Fig. S1. NMR structure and dynamics analysis of the TAM domain

a. ^{15}N R_1, R_2 relaxation rates, $\{^1H\}$-^{15}N heteronuclear NOEs and ^{13}C secondary chemical shifts recorded on TIP5/TAM versus residue numbers. The sequence of the TAM domain and the secondary structure elements are indicated on top. Amides that are solvent-protected and show reduced H/D exchange are indicated by green circles. NMR signals that remain observable in SOFAST-HMQC spectra after 90 min are indicated by dark green circles. Error bars for the relaxation data fit and $\{^1H\}$-^{15}N heteronuclear NOE were calculated as described in Materials and Methods. Based on the ratio of ^{15}N R_2/R_1 relaxation rates the tumbling correlation time (τ_c) of the TAM domain corresponds to a monomer in solution (τ_c = 9-10 ns, compared to τ_c = 9.7 ns expected for a 113 residue monomeric protein, see NMR methods).

b. Stereo view of the NMR ensemble of the 20 lowest energy structures. Secondary structure elements are colored green.

Supplementary Fig. 2

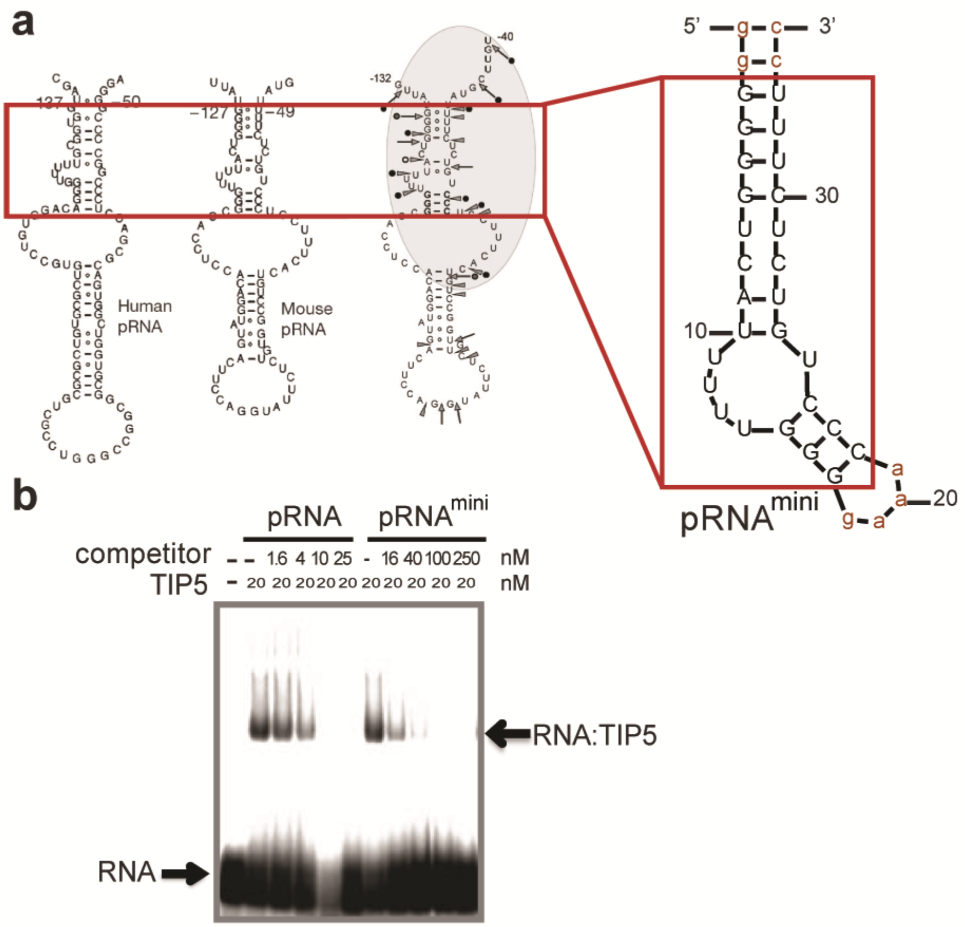

Suppl. Fig. S2. TIP5 binds to pRNA^{mini}

a. Predicted secondary structure of human and mouse pRNA supported by RNase footprinting (1). Arrows and arrow heads indicate cleavage sites of RNase T1 and RNase V1, respectively. Cleavage sites that are protected by TIP5 binding are highlighted by black circles. The region of pRNA that is shielded from cleavage is shaded grey. The secondary structure shared by human and mouse pRNA that is recognized by TIP5 is contained in pRNA^{mini} (red box).

b. Electrophoretic mobility shift assay (EMSA) comparing binding of TIP5 to pRNA or pRNA^{mini}. Murine TIP5 (residues 1-598) was preincubated with radiolabeled non-specific RNA in the presence of increasing amounts of unlabeled pRNA or pRNA^{mini} . Arrows indicate positions of the free RNA probe and RNA:TIP5 complexes.

Supplementary Fig. 3

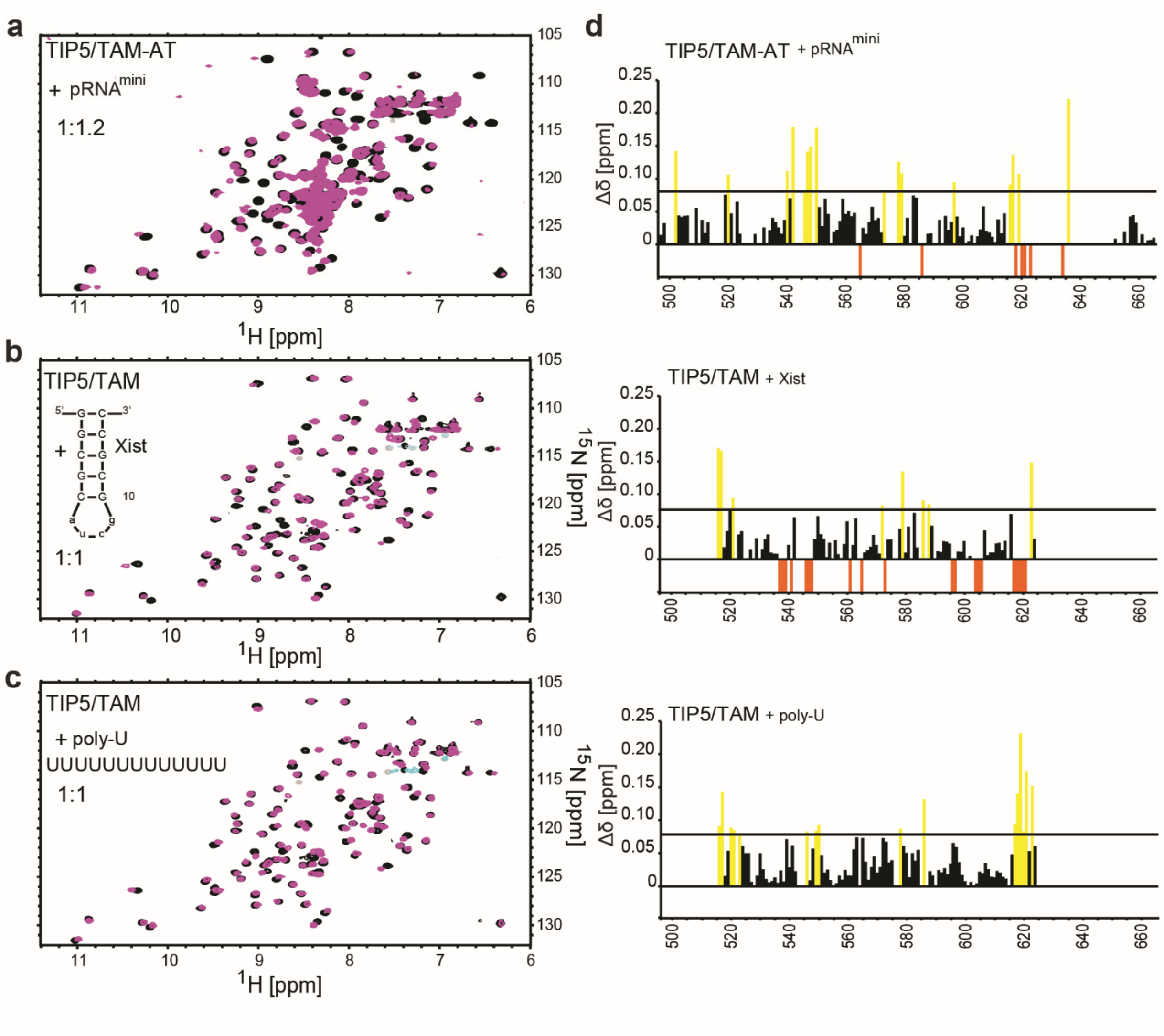

Suppl. Fig. S3. TIP5/TAM titrations with short RNAs

a. Overlay of ^{1}H,^{15}N HSQC spectra of ^{15}N labeled TIP5/TAM-AT free (black) and bound to 1.2 molar amounts of pRNA^{mini} (magenta).

b. Overlay of ^{1}H,^{15}N HSQC spectra of ^{15}N labeled TIP5/TAM free (black) and bound to equimolar amounts of an Xist RNA A repeat hairpin (magenta, see insert) (2).

c. Overlay of ^{1}H,^{15}N HSQC spectra of ^{15}N labeled TIP5/TAM free (black) and at equimolar ratio of a single-stranded 13-mer poly-U RNA (magenta, see insert).

d. Chemical shift perturbation vs. residue number upon binding of TIP5/TAM-AT to pRNA^{mini} (top), TIP5/TAM to the Xist RNA hairpin (middle) and TIP5/TAM to poly-U RNA (bottom). NMR signals that are exchange-broadened are colored orange. Residues with chemical shift changes greater than one standard deviation from Δδ = 0.078 ppm (average value for pRNA^{mini} titration) are colored yellow.

Supplementary Fig. 4

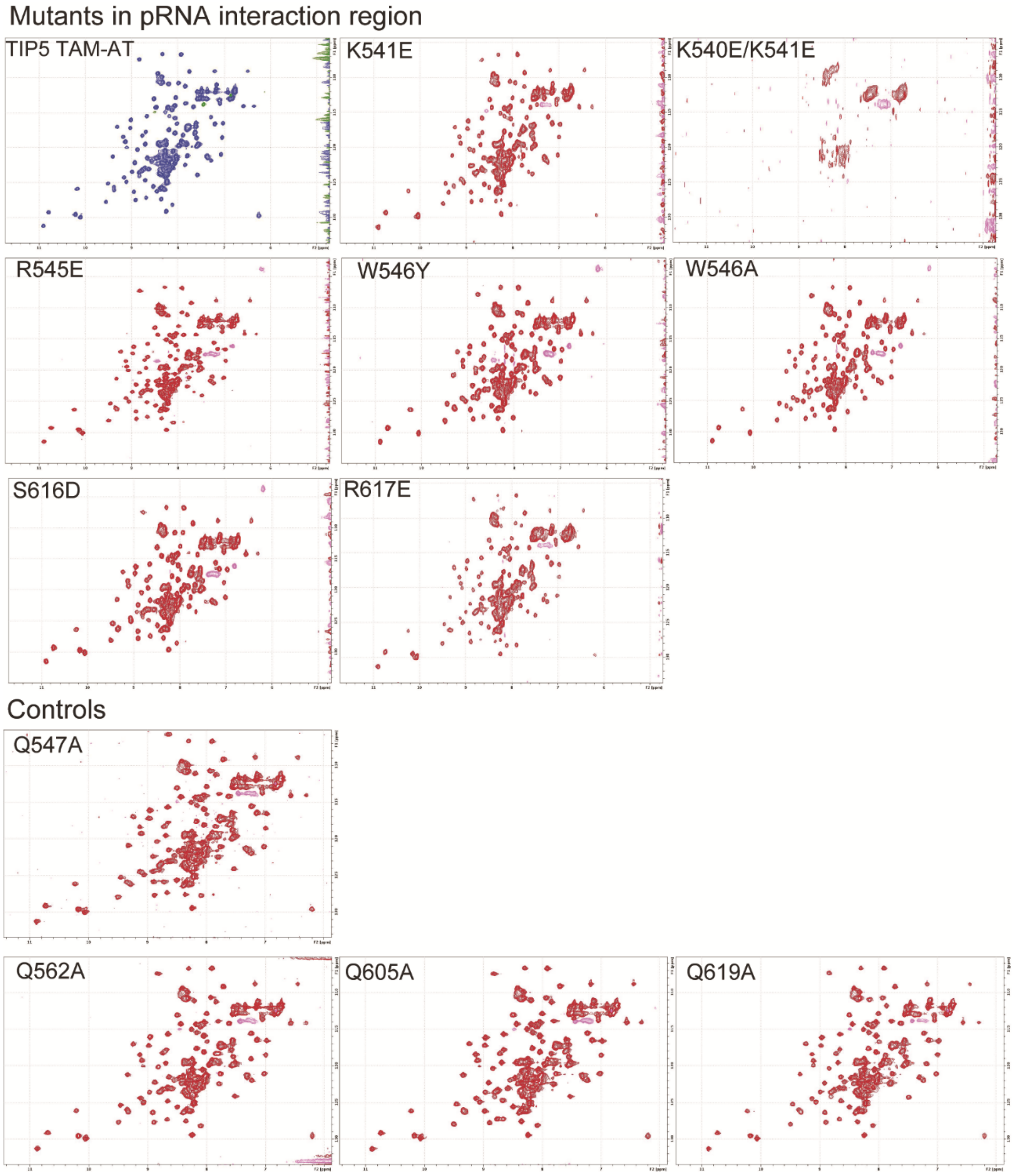

Suppl. Fig. S4. ^{1}H,^{15}N HSQC spectra of ^{15}N-labeled TIP5/TAM-AT wild type and mutants

Comparison of the spectra indicates that all proteins adopt a globular fold with the exception of the K540E/K541E double-mutant, which is misfolded and/or aggregated at the buffer conditions tested.

Supplementary Fig. 5

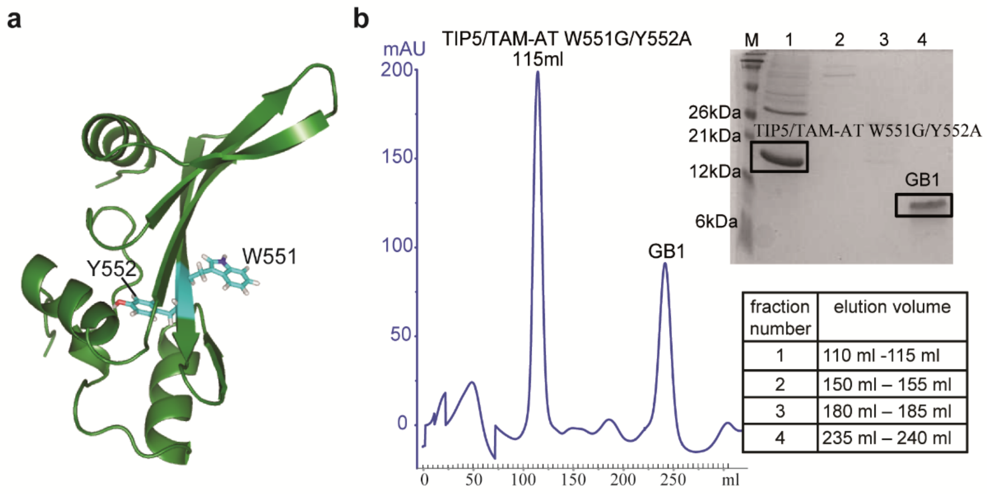

Suppl. Fig. S5. The TIP5/TAM-AT W551G/Y552A protein is unstable

a. The side chains of W551 and Y552 are shown in the structure of the human TAM domain. These residues contribute to the core of the domain.

b. TIP5/TAM-AT W551G/Y552A elutes in the void volume on a Superdex S75 HiLoad 26/60 gel filtration column. Elution profile and an SDS-PAGE of selected elution fractions with bands corresponding to TIP5/TAM-AT W551G/Y552A and the GB1 expression tag of the same construct. The fractions numbers indicated above the lanes correspond to the numbers in the elution profile. Note that, the folded wildtype TIP5/TAM-AT protein elutes on the same column at 170 ml (data not shown).

Supplementary Fig. 6

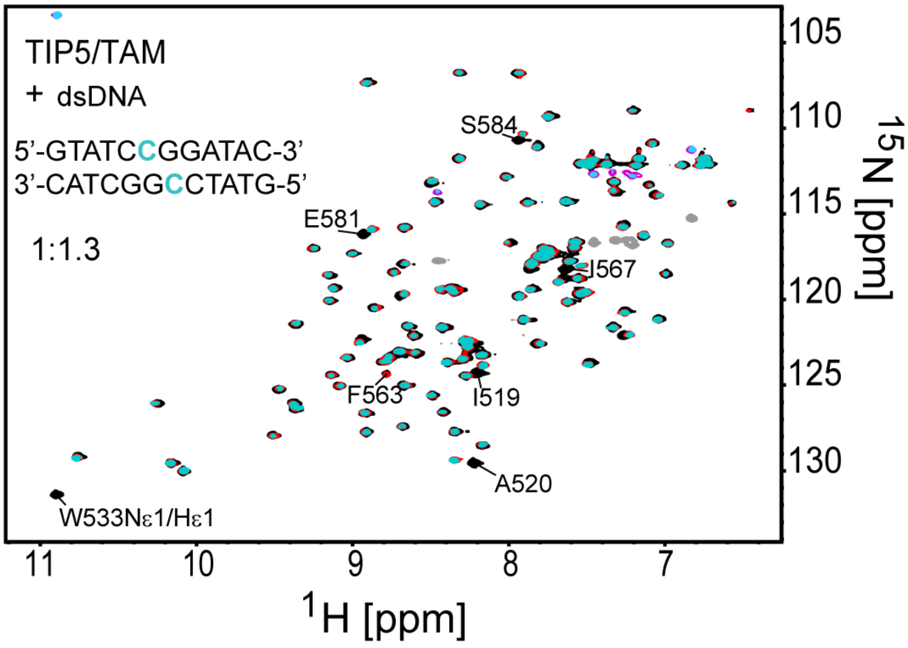

Suppl. Fig. S6. DNA binding of the TIP5/TAM domain

Overlay of ^{1}H,^{15}N HSQC spectra of ^{15}N labeled TIP5/TAM free (black) and in the presence of 1.3 fold molar excess of unmethylated double-stranded DNA oligonucleotide (5'-GTATCCGGATAC-3', red) or the same oligonucleotide methylated at C5 (cyan), which is recognized by human MBD1 (3). Some residues that show minor chemical shift changes are indicated. The DNA duplex is shown in the insert.

Supplementary References

1. Mayer, C., Neubert, M. and Grummt, I. (2008) The structure of NoRC-associated RNA is crucial for targeting the chromatin remodelling complex NoRC to the nucleolus. *EMBO Rep*, **9**, 774-780.
2. Duszczyk, M.M., Wutz, A., Rybin, V. and Sattler, M. (2011) The Xist RNA A-repeat comprises a novel AUCG tetraloop fold and a platform for multimerization. *RNA*, **17**, 1973-1982.
3. Ohki, I., Shimotake, N., Fujita, N., Jee, J., Ikegami, T., Nakao, M. and Shirakawa, M. (2001) Solution structure of the methyl-CpG binding domain of human MBD1 in complex with methylated DNA. *Cell*, **105**, 487-497.
